# A comparative study of eight serological methods shows that spike protein-based ELISAs are the most accurate tests for serodiagnosing SARS-CoV-2 infections in cats and dogs

**DOI:** 10.3389/fvets.2023.1121935

**Published:** 2023-01-26

**Authors:** Carlos Diezma-Díaz, Gema Álvarez-García, Javier Regidor-Cerrillo, Guadalupe Miró, Sergio Villanueva-Saz, María Dolores Pérez, María Teresa Verde, Patricia Galán-Malo, Alejandro Brun, Sandra Moreno, Rocío Checa, Ana Montoya, Wesley C. Van Voorhis, Luis Miguel Ortega-Mora

**Affiliations:** ^1^SALUVET, Animal Health Department, Faculty of Veterinary Sciences, Complutense University of Madrid, Ciudad Universitaria s/n, Madrid, Spain; ^2^SALUVET-Innova S.L., Faculty of Veterinary Sciences, Complutense University of Madrid, Madrid, Spain; ^3^PetParasiteLab, Animal Health Department, Faculty of Veterinary Sciences, Complutense University of Madrid, Ciudad Universitaria s/n, Madrid, Spain; ^4^Clinical Immunology Laboratory, Department of Animal Pathology, Faculty of Veterinary Sciences, Instituto Agroalimentario de Aragón (IA2), Zaragoza University and Agro-food Research and Technology Centre of Aragon, Zaragoza, Spain; ^5^Food Technology, Faculty of Veterinary Sciences, AgriFood Institute of Aragón (IA2) Zaragoza University and Agro-food Research and Technology Centre of Aragon, Zaragoza, Spain; ^6^ZEULAB S.L., Zaragoza, Spain; ^7^Animal Health Research Centre, Spanish National Institute for Agricultural and Food Research and Technology/Spanish National Research Council (INIA/CSIC), Madrid, Spain; ^8^Department of Medicine, Division of Allergy and Infectious Diseases, Center for Emerging and Re-emerging Infectious Diseases, University of Washington, Seattle, WA, United States

**Keywords:** SARS-CoV-2 virus, cat, dog, serological tests, RBD fragment, nucleocapsid protein

## Abstract

**Introduction:**

Coronavirus disease 2019 (COVID-19) is an infectious zoonotic disease caused by SARS-CoV-2. Monitoring the infection in pets is recommended for human disease surveillance, prevention, and control since the virus can spread from people to animals during close contact. Several diagnostic tests have been adapted from humans to animals, but limited data on the validation process are available.

**Methods:**

Herein, the first comparative study of six “*in house*” and two commercial serological tests developed to monitor SARS-CoV-2 infection in pets was performed with a well-coded panel of sera (61 cat sera and 74 dog sera) with a conservative criterion (viral seroneutralisation and/or RT–qPCR results) as a reference. Four “*in house*” tests based on either the RBD fragment of the spike protein (RBD-S) or the N-terminal fragment of the nucleoprotein (N) were developed for the first time. The analytical specificity (ASp) of those tests that showed the best diagnostic performance was assessed. The validation included the analysis of a panel of sera obtained pre-pandemic from cats and dogs infected with other coronaviruses to determine the analytical Sp (17 cat sera and 41 dog sera).

**Results and discussion:**

ELISAS based on the S protein are recommended in serosurveillance studies for cats (RBD-S SALUVET ELISA, ELISA COVID UNIZAR and INgezim^®^ COVID 19 S VET) and dogs (INgezim^®^ COVID 19 S VET and RBD-S SALUVET ELISA). These tests showed higher diagnostic sensitivity (Se) and DSp in cats (>90%) than in dogs. When sera obtained prior to the pandemic and from animals infected with other coronaviruses were analyzed by RBD-S and N SALUVET ELISAs and INgezim^®^ COVID 19 S VET, a few cross reactors or no cross reactions were detected when dog and cat sera were analyzed by tests based on the S protein, respectively. In contrast, the number of cross reactions increased when the test was based on the N protein. Thus, the use of tests based on the N protein was discarded for serodiagnosis purposes. The results obtained revealed the most accurate serological tests for each species. Further studies should attempt to improve the diagnostic performance of serological tests developed for dogs.

## 1. Introduction

Coronavirus disease 2019 (COVID-19) is a zoonotic infectious disease caused by the SARS-CoV-2 virus. The emergence of SARS-CoV-2 was first observed when cases of unexplained pneumonia were noted in the city of Wuhan, China (1). The COVID-19 virus was then rapidly isolated from patients and sequenced. SARS-CoV-2 is a positive-stranded RNA virus belonging to the Coronaviridae family (a betacoronavirus subgroup B) with high homology with that of the coronavirus that caused SARS in 2002–2004 (2). Since then, COVID-19 has caused nearly 6.5 million human deaths and unprecedented global impacts (WHO-COVID-19 Weekly Epidemiological Update November 16th, 2022).

An increasing number of animal species are susceptible to SARS-CoV-2 natural infections (ferret, cat, dog, mink, tiger, lion, gorilla, puma, and snow leopard) (3–5). However, genetic and epidemiological studies have suggested that these infections were introduced from humans rather than enzootic virus circulation (WHO-convened Global Study of Origins of SARS-CoV-2: China Part OMS). Experimental infections have also demonstrated the susceptibility of several animals to SARS-CoV-2 infection, mainly cats, ferrets, hamsters, and deer, as well as a certain resistance to infection by other species (cattle, pigs, chickens) (5, 6).

In particular, the role of cats, dogs, and hamsters in the transmission of the disease has been studied in detail due to the close contact of pets with humans. Several studies agree that cats are more susceptible than other species; they may show vague signs of respiratory illness or suffer a subclinical infection without clinical signs. Infections are usually associated with pets that have been in contact with infected owners (6–8). Currently, cats and dogs are not considered the origin for human infection, but prevention and control of COVID-19 might benefit from clarifying the role of pets in the epidemiology of the disease and new SARS-CoV-2 variants (6, 9). Thus far, we are still in need of diagnostic tests suitable for SARS-CoV-2 infection surveillance.

Several serological techniques used in humans (10) have been adapted to monitor animal exposure to SARS-CoV-2 in pets. Indirect ELISAs based on the nucleocapsid protein (N) (11–13) and the RBD fragment of the spike protein (S) (6, 13, 14) have been developed and are frequently used in seroprevalence studies. However, there is a lack of previous validation studies on serological methods in pets according to OIE standards, including the assessment of their analytical and diagnostic performance. Cross-reactions between SARS-CoV-2 and other pathogens are suspected based on the scenario described for humans (15). Moreover, a well-coded panel of reference sera is needed. In this regard, the viral seroneutralisation assay (VNA) has been frequently employed as a confirmatory test or as a reference test for ELISA standardization with limitations since only positive or doubtful sera were tested (11, 14).

Herein, a comparative study between six “*in house*” serological techniques and two commercial tests developed for monitoring SARS-CoV-2 exposure in pets (cats and dogs) was performed. For the validation process, viral seroneutralisation and/or detection of virus elimination by RT–qPCR at least 21 days prior to sera collection were employed as reference criteria, and a “well-coded” panel of sera was selected and analyzed by all serological tests. Also, a panel of prepandemia sera was employed to determine analytical specificity (ASp). The final aim was to reveal the most accurate serological test for each species in surveillance studies.

## 2. Materials and Methods

### 2.1. Experimental design

A “well-coded” panel of sera (Table 1; see Section 2.2.1) was employed to determine the diagnostic performance of all evaluated tests (Table 2) with a conservative criterion (viral seroneutralisation titers equal or higher that 1/20 and/or virus detection by RT–qPCR at least 21 days prior to sera collection as a reference). Tests were classified by the antigen-N- or S-viral protein- and by the technique-ELISA, Western blot or lateral flow immunoassay (LFIA)-employed (Table 2). The analytical specificity (ASp) of the tests with the best diagnostic performance was evaluated for both proteins and for both species with the sera described in Section 2.2.2. Due to the limited volumes of these sera, only the ASp of the “*in house*” N and RBD-S SALUVET ELISAs, and INgezim^®^ COVID19 S VET was analyzed, and precision was estimated for N and RBD-S SALUVET ELISAs.

**Table 1:** Reference sera employed.

**Cat sera**										**Dog sera**									
**Analytical specificity**		**Diagnostic performance**								**Analytical specificity**		**Diagnostic performance**							
***n*** = **17**		***n*** = **61**								***n*** = **41**		***n*** = **74**							
**Prepandemic**	**Other coronaviruses** ^a^	**Veterinary clinics**						**Shelters**		**Prepandemic**	**Other coronaviruses** ^b^	**Veterinary clinics**						**Shelters**	
***n*** = **13**	***n*** = **4**			***n*** = **44**				***n*** = **17**		***n*** = **36**	***n*** = **5**			***n*** = **71**				***n*** = **3**	
				VNA				VNA						VNA				VNA	
				+	–			+	–					+	–			+	–
		RT-qPCR	+	1	0	RT-qPCR	+	0	0			RT-qPCR	+	1	1	RT-qPCR	+	0	0
			–	10	33		–	1	16				–	10	59		–	0	3

^a^Sera from cats diagnosed with feline infectious peritonitis (FIP).

^b^Sera from dogs vaccinated with VANGUARD^®^ PLUS CPV/CV (Zoetis).

VNA, virus neutralization assay.

+, positive result. Sera were collected at least 21 days after obtaining a RT-qPCR positive result from nasal or oropharyngeal swaps.

–, negative result.

**Table 2:** Serological tests evaluated in the comparative study to monitor SARS CoV-2 infection in pets.

**Test**	**Comercial/*In house***	**Type of technique**	**Antigen**	**Cut-off**	**Sensitivity (DSe)**	**Specificity (DSp)**	**References**
RBD-S SALUVET ELISA	*In house*	ELISA	RBD- S protein	Cat: RIPC > 17.58	**Cat: 100%**	**Cat: 94%**	This work
				Dog: RIPC > 31	Dog: 60%	Dog: 95%	
RBD-S SALUVET Western blot	*In house*	Western blot	RBD- S protein	Protein recognition (positive/negative)	Cat: 72%	Cat: 96%	This work
					Dog: 50%	Dog: 93%	
LFIA COVID UNIZAR	*In house*	LFIA	RBD-S protein	Qualitative result (positive/negative)	Cat: 72%	Cat: 100%	This work
					Dog: 9%	Dog: 98%	
ELISA COVID UNIZAR	*In house*	ELISA	RBD- S protein	OD sample ≥ 0.47	**Cat: 90%**	**Cat: 98%**	This work
					Dog: 9%	Dog: 98%	
INgezim^®^ COVID19 S VET	Commercial	ELISA	S-protein	OD sample > OD negative control + 0.25 (Cat) or + 0.35 (Dog)	**Cat: 100%**	**Cat: 96%**	Manufacturer's instructions
					Dog: 82%	Dog: 83%	
N-SALUVET ELISA	*In house*	ELISA	N-terminal N Protein	Not established^*^	–	–	This work
N -SALUVET Western blot	*In house*	Western blot	N-terminal N Protein	Protein recognition (positive/negative)	Cat: 63 %	Cat: 77%	This work
					Dog: 25%	Dog: 98%	
ID Screen^®^ SARS-CoV-2	Commercial	ELISA	N- Protein	>60 S/P (sample/negative)	Cat: 63%	Cat: 96%	Manufacturer's instructions
					Dog: 36%	Dog: 85%	

LFIA, lateral flow immunoassay; RIPC, relative index percent; S/P, sample positive ratio; OD,optical density.

INgezim^®^ COVID19 S VET (Eurofins, INGENASA, Spain); ID Screen^®^ SARS-CoV-2 (IDVet, France).

The highest DSe and DSp values are marked in bold letters.

^*^No acceptable values for DSe and DSp were reached at any cut-off.

### 2.2. Sera panels

#### 2.2.1. Reference sera employed to determine diagnostic performance

Sixty-one cat and 74 dog sera from different veterinary clinics and animal shelters located in the Community of Madrid (Spain) were tested by different serological techniques for the detection of specific anti-SARS-CoV-2 antibodies. RT–qPCR results were available for each individual animal (Table 1). Samples came from an epidemiological study carried out in the Community of Madrid throughout 2021, coinciding with the third and fourth waves of COVID-19 in Madrid (INE, https://www.ine.es/covid/covid_inicio.htm; Accessed in June 2022). Owner's consent was obtained in accordance with the Spanish Animal Protection laws and International Guiding Principles for Biomedical Research Involving Animals issued by the Council for International Organizations of Medical Sciences.

For each animal, a nasopharyngeal and/or an oropharyngeal swab was collected in inactivated virus transport and preservation medium tubes (Biocomma, Durviz, Valencia, Spain) for SARS-CoV-2 testing. Blood samples were also drawn by cephalic venipuncture in accordance with good clinical practices. Swabs were analyzed by RT–qPCR (see Section 2.3.1). Sera were transported to the laboratory for heat inactivation for 1 h at 56°C and stored at −20°C until analysis (8). Animals were resampled periodically for RT–PCR SARS-CoV-2 detection. Sera for analyses were collected starting at 15 days after RT–PCR SARS-CoV-2 detection from swabs.

#### 2.2.2. Reference sera employed to determine the ASp of tests with the best diagnostic performance

Thirteen cat and 36 dog sera collected before the SARS-CoV-2 pandemic (named prepandemic pet sera) were included in the study. These sera came from a biobank provided by PetParasiteLab composed of sera from healthy pets or animals with other non-coronavirus-related diseases, mainly vector-borne diseases such as leishmaniosis.

Moreover, four cat sera from animals that died from feline infectious peritonitis (FIP) at the Veterinary Hospital—Complutense University of Madrid (VCH–UCM). Diagnosis of FIP was confirmed by histopathology in target tissue samples obtained post-mortem after humanitarian euthanasia. Cats were euthanized by intravenous administration of 133 mg sodium pentobarbital/kg bw (equivalent to 1 ml/1.5 kg) (Dolethal 200 mg/ml, Vetoquinol, Spain). Five sera from dogs vaccinated with VANGUARD^®^ PLUS CPV/CV (Zoetis, New Jersey, USA), which consists of canine parvovirus strain NL-35-D > 10^7, 2^ TCID_50_ (tissue culture infective dose) and canine coronavirus strain NL-18 ≥ 1.49 RP (relative potency), were also included.

### 2.3. Reference assays

#### 2.3.1. SARS-CoV-2 real time PCR detection

Basically, the procedure previously described by Miró et al. (8) was followed. Viral RNA was obtained from 200 μl of nasal and oropharyngeal swabs transport medium using the Maxwell R RSC Viral Total Nucleic Acid Purification Kit for automated extraction in a Maxwell R RSC 48 Instrument (Promega, Madrid, Spain). A Multiplex TaqPath™ COVID-19 CE-IVD RT–PCR Kit targeting the SARS-CoV-2 orf1-ab, S and N genes (Applied Biosystems, Spain) was performed according to the manufacturer's instructions for swabs.

#### 2.3.2. Virus seroneutralisation assay

For VNA, a protocol as described by Amanat et al. (16) with modifications was followed. Briefly, a fixed amount of SARS-CoV-2 virus (BetaCoV/Netherlands/01 strain kindly provided by Dr. R. Molenkamp—Erasmus Medical Center, Rotterdam, Netherlands) inoculum (1,600 pfu) causing complete cytopathic effect (CPE) in 48 h was incubated with heat-inactivated cat or dog serum samples that were 3-fold serially diluted in Dulbecco's modified Eagle's medium (DMEM) (ThermoFisher Scientific, Waltham, Massachusetts, USA) containing supplements (10% fetal bovine serum, 2 mM glutamine, 100 U/mL penicillin, 100 mg/mL streptomycin). The assay was performed in duplicate starting at 1:20 dilution. After 1 h of incubation at room temperature, the serum-virus mixtures were added to semiconfluent Vero E6 cell monolayers (ATCC/CRL/1586) seeded onto 96-well plates and incubated for 1 h at 37°C in a 5% CO_2_ incubator. After incubation, the supernatants were removed, and fresh serum sample dilutions were added to the plate wells. The cells were maintained for an additional 48 h until CPE was evident in infected control wells (without serum samples). The cells were then fixed with 10% formaldehyde and stained with 2% crystal violet solution. Determination of the neutralization titer was expressed as the highest dilution of serum in which no CPE was observed.

### 2.4. Evaluated serological assays

As shown in Table 2, two commercial and six “*in-house*” serological methods were evaluated to detect SARS-CoV-2 infection in cats and dogs.

#### 2.4.1. RBD-S and N SALUVET ELISAs

The IgG antibody against the SARS-CoV-2 spike-receptor binding domain (RBD-S) or the nucleoprotein N-terminal RNA binding domain in serum samples was tested using an enzyme-linked immunosorbent assay (RBD-S and N SALUVET ELISAs, respectively). The RBD-S fragment was produced in HEK293 mammalian cells (transients) from the Institute for Protein Design, Seattle, WA as previously described by Phan et al. (17). The nucleoprotein N-terminal (RNA binding domain) [N–N term] fragment that comprises the sequence between aa 47 and aa 173 has an estimated Mw of 16.62 kDa. It was produced in *E. coli* and purified by Ni chromatography and size exclusion chromatography to a single band on SDS–PAGE in Wes Van Voorhis's laboratory (University of Washington, Seattle, United States).

RBD-S ELISA was performed as previously described by Phan et al. (17) for humans with modifications. Based on the evaluation of different protein concentrations per well, blocking solutions and secondary antibodies for each species until the highest ratio (average OD positive control/OD negative control) was obtained. Positive control serum showed seroneutralisation titres > 180 (as the highest dilution without CPE), and negative control serum was negative by VNA (titter < 20).

One hundred microlitres of coating buffer containing S-RBD at 1 μg/mL produced was used to coat the microtitre plates (ImmunoPlate Maxisorp, Nunc, Roskilde, Denmark) overnight at 4°C. After three washes with phosphate-buffered saline containing 0.05% Tween 20 (PBST), the blocking step was performed with PBST containing 1% casein sodium salt from bovine milk (Merck, Darmstadt, Germany) for 1 h at 37°C, followed by three washes with PBST. Next, 100 μl of cat or dog sera diluted 1:80 was added and incubated for 1 h at 37°C. The specific IgG response was revealed using either a peroxidase goat anti-cat IgG (H + L) conjugate (Jackson ImmunoResearch, Cambridge, UK) or peroxidase rabbit anti-dog IgG (H + L) conjugate (Jackson ImmunoResearch, Cambridge, UK), both diluted 1:5,000 in PBST. The antibodies were detected using 2,2′-Azino-bis (3-ethylbenzothiazoline-6-sulfonic) acid substrate (ABTS) (Merck, Darmstadt, Germany). Absorbance was measured at 405 nm using a microplate reader (Multiscan RC 6.0, Labsystems). Optical density (OD) values samples were converted into a relative index percent (RIPC) by employing the formula: RIPC = (OD405 sample – OD405 – C)/(OD405 + C – OD405 – C) × 100. The positive control reached an OD value of 0.9–1 within 20 min after ABTS addition. The negative control showed OD < 0.2 after adding the stop solution (oxalic acid 0.1 M).

N SALUVET ELISA was performed in the same way as RBD-S SALUVET ELISA.

#### 2.4.2. ELISA COVID UNIZAR

An *in-house* indirect ELISA for the detection of IgG specific for RBD-S was established. Ninety-six-well plates were coated overnight at 4°C with 100 ng RBD-S protein in phosphate buffered saline (PBS). Subsequently, the coating solution was removed, and the plate was washed three times with 200 μL per well of PBS + TWEEN 0.05% (PBST). A volume of 300 μL of PBST containing 3% dry skimmed milk (PBST-M) was added to each well as a blocking solution and incubated for 1 h at 37°C in a humidified chamber. Then, 100 μL of cat or dog sera diluted 1:100 in PBST-M was added to each well and incubated for 1 h at 37°C in a humidified chamber. After washing the plates for 30 s 6 times with PBST followed by 1 wash with PBS for 1 min, 100 μL/well of multi-species horseradish peroxidase conjugate (ThermoFisher Scientific, Waltham, Massachusetts, USA) diluted 1:100,000 in PBST-M was added per well and incubated for 1 h at 37°C in the moist chamber and washed again with PBST and PBS as described above. The substrate solution (ortho-phenylene-diamine) and stable peroxide substrate buffer (ThermoFisher Scientific, Waltham, Massachusetts, USA) were added at 100 μL per well and developed for 20 ± 5 min at room temperature in the dark. The reaction was stopped by adding 100 μL of 2.5 M H_2_SO_4_ to each well. Absorbance values were read at 492 nm in an automatic microELISA reader (ELISA Reader Labsystems Multiskan, Midland, Canada). As positive controls, each plate included two serum samples of human patients diagnosed with COVID-19, which were confirmed by a molecular test and a commercial quantitative ELISA, and two serum samples from a seropositive cat and a seropositive dog to SARS-CoV-2. The same positive and negative sera were used in all assays. All samples were run in duplicate. The cut-off was set to 0.47 OD units. The results above this value were considered positive.

#### 2.4.3. INgezim^®^ COVID 19 S VET

INgezim^®^ COVID 19 S VET (Eurofins, INGENASA, Spain) was used following the manufacturer's instructions. This indirect ELISA uses the S protein as an antigen and the peroxidase-conjugated recombinant protein A/G as a conjugate. The use of this test is recommended for serum and plasma samples from minks, ferrets, cats, and dogs. Cut-off was established by the manufacturer (positive cat serum: OD sample > OD negative control + 0.25; positive dog serum: OD sample > OD negative control + 0.35).

#### 2.4.4. ID Screen^®^ SARS-CoV-2 double antigen multispecies ELISA

ID Screen^®^ SARS-CoV-2 Double Antigen ELISA (IDVet, rue Louis Pasteur, Grabels, France) was used following the manufacturer's instructions. This double-antigen ELISA is based on the detection of antibodies directed against purified recombinant N protein. Test results were expressed as sample/positive control (S/P %) ratios based on positive and negative controls. Sera with S/P ratios < 50% were considered negative, between 50 and 60% were doubtful and ≥60% were positive.

#### 2.4.5. RBD-S and N SALUVET Western blots

RBD-S and nucleoprotein N-terminal (N) proteins (10 μg/membrane) were mixed with bromophenol blue (Bio-Rad Laboratories, CA, USA) (2X) and 1,4-dithioerythreitol (DTE) (2%) and then boiled for 5 min. Electrophoresis was performed in 12% polyacrylamide gels and then electrotransferred to a nitrocellulose membrane for Western blotting (Mini Trans-Blot Cell, Bio-Rad Laboratories) (18). Membranes were washed in Tris–phosphate-buffered saline with 0.05% Tween-20 (TBS-T) and then incubated for 1 h at 37°C in blocking buffer [TBS-T, containing 1% casein sodium salt from bovine milk (Merck, Darmstadt, Germany)]. Next, the membranes were incubated with cat or dog sera at a 1:20 dilution in blocking buffer for 1 h at 37°C. After three washes with TBS-T for 10 min each, the membranes were incubated with the secondary antibodies employed in Section 2.4.1 and diluted 1:500 in TBS-T for 1 h at 37°C. Finally, three 10 min washes with TBS-T were performed, and the reaction was developed using 4-chloro-1-naphtol (Bio-Rad Laboratories) as substrate. Detection of the protein band with suitable molecular weight (RBD-S: 26.54 Kd, N-terminal nucleoprotein: 16.62 Kd) was considered a positive result (Supplementary Figure).

#### 2.4.6. LFIA COVID UNIZAR

Gold nanoparticles of 40 nm (Abcam, Cambridge, UK) were bound to RBD-S or anti-ovoalbumin antibodies. RBD-S and antibodies at a concentration of 0.01 mg/mL in 20 mM carbonate buffer at pH 10.9 were incubated with particles overnight at room temperature. Then, 10% blocking solution (ZEULAB, S.L.) was added to the suspension and incubated at room temperature for 2 h. After centrifugation at 20,000 × *g* for 15 min, the coated beads were resuspended in washing buffer (ZEULAB, S.L.). Finally, beads conjugated with RBD, or anti-ovalbumin antibodies were mixed at a ratio of 1:1 and dispensed over the conjugate pad of a glass fiber membrane (GE Healthcare) using a ZX 1010 Dispenser (Bio-Dot, Irvine, USA).

RBD-S for the test line and ovalbumin for the control line were sprayed onto a nitrocellulose membrane at a concentration of 0.5 mg/mL using a ZX 1010 dispenser (Bio-Dot, Irvine, USA). The conjugate, nitrocellulose membrane, and adsorbent pads were assembled on a baking card, and strips of 4 mm width were cut using a CM4000 guillotine cutter (Bio-Dot, Irvine, USA). The test procedure was performed by dipping the strip into 30 μL of serum samples diluted in 150 μL of analysis buffer (ZEULAB, S.L.) and incubated for 10 min. If only the control band (upper band) was developed, the result was considered negative. When two bands developed, the result was considered positive. If the control band did not appear, the result was invalid.

### 2.5. Data analysis

A non-parametric two-graph receiver operating characteristic (TG-ROC) analysis using SigmaPlot software was applied for all tests. Area under the curve (AUC), diagnostic sensitivity (DSe) and specificity (DSp) values were calculated for each assay by employing VNA and RT-qPCR results as thereference criterium. A serum was considered as a reference positive serum when the animal of origin showed either a RT-qPCR positive result, a SN positive result or both RT-qPCR and SN positive results, considering 1/20 SN titer as the cut-off. Cut-off values for the RBD-S and N SALUVET ELISAs developed for cats and dogs were established. Test agreement values [expressed as Kappa *(k)*-values] between techniques were calculated using WinEpiscope (http://www.winepi.net/). To assess the correlation between tests, the Pearson correlation coefficient (Pearson r) was calculated with GraphPad Prism 6.01 software (San Diego, CA, USA).

The precision of the RBD-S and N SALUVET ELISAs was measured by estimating intra-assay and inter-assay repeatability. Twenty cat sera (10 positive and 10 negative sera by VNA) and 13 dog sera (six positive and seven negative sera by VNA) were run in triplicate. The coefficients of variation (CVs) [(standard deviation of the replicates/mean of replicates) × 100] were calculated using the raw absorbance values. Coefficients of variation, with values < 20% for raw absorbance values, indicated adequate repeatability (19).

## 3. Results

### 3.1. Serological tests developed for cats

#### 3.1.1. Diagnostic performance of all serological tests

**Figure 1:**
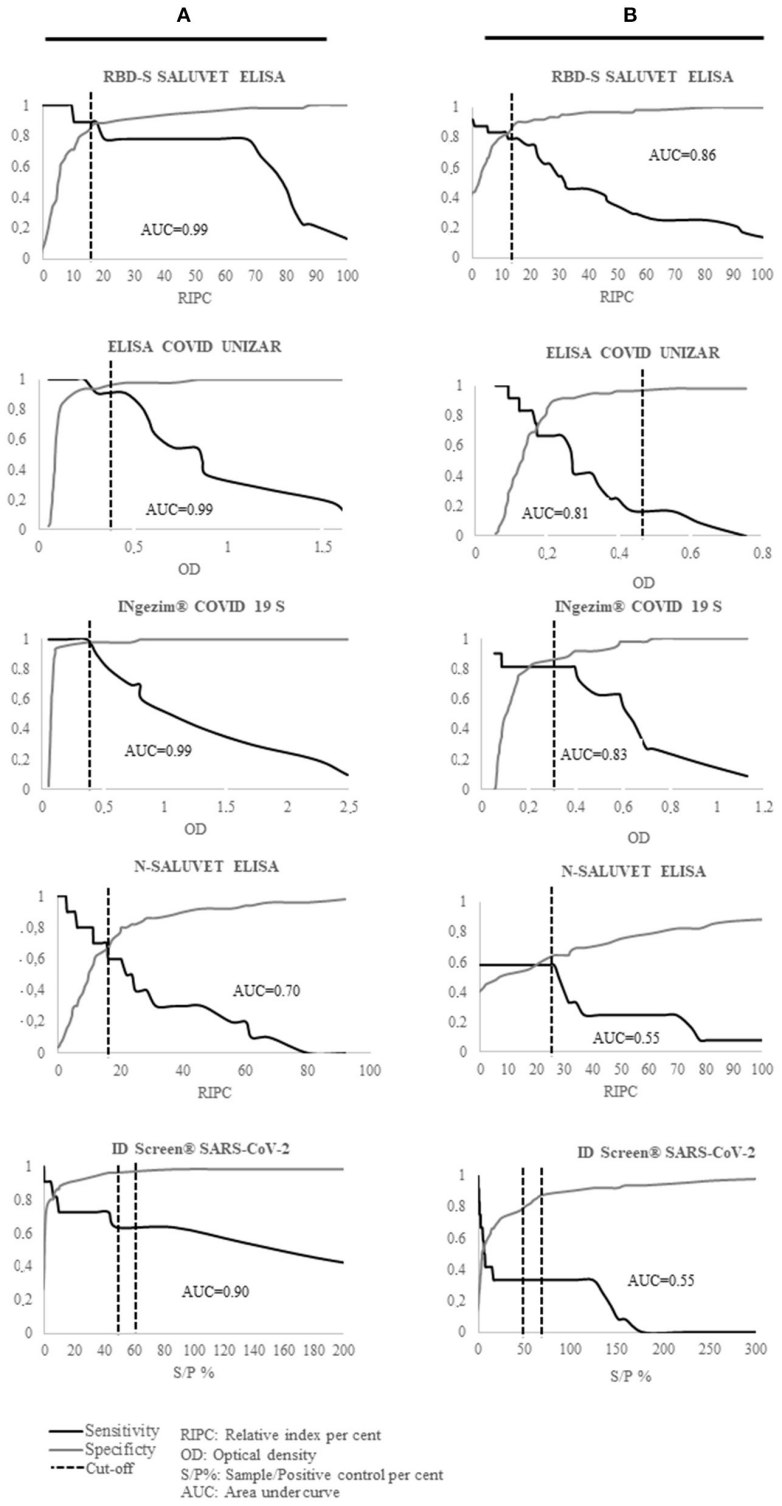
Two-graph receiver operating characteristics (TG-ROC) analyses based on the reference criterion (VNA and PCR results) and the area under the curve (AUC) values shown by all serological tests developed for cats **(A)** and dogs **(B)**. S/P%, sample/positive control percent; AUC, area under curve.

The AUC, and DSe and DSp values are shown in Figure 1A and Table 2, respectively.

RBD-S SALUVET ELISA, ELISA COVID UNIZAR and INgezim^®^ COVID 19 S VET showed excellent performance with an AUC of 0.99 (RBD-S SALUVET ELISA 95% confidence interval CI: 0.975–1.007; ELISA COVID UNIZAR CI: 0.967–1.008; INgezim^®^ COVID 19 S VET CI: 0.980–1.007). RBD-S SALUVET ELISA showed 100% Se (95% CI: 0.715–1) and 94% Sp (95% CI: 0.835–0.988) for a cut-off with a RIPC value of 17.58.

The N SALUVET ELISA showed an AUC = 0.70 (95% CI: 0.508–0.885), and the cut-off was established as 15.44 with 70% Se (95% CI: 0.347–0.933) and 66% Sp (95% CI: 0.512–0.787). An AUC value of 0.90 corresponded to ID Screen^®^ SARS-CoV-2 (CI: 0.763–1.020).

#### 3.1.2. Test agreement and correlation

As shown in Table 3A, tests based on the detection of specific anti-S-protein antibodies showed higher agreement than tests based on N-protein. The reference criterium showed almost perfect agreement (*k* > 0.80) with S-RBD ELISA, ELISA COVID UNIZAR, LFIA COVID UNIZAR and INgezim^®^ COVID 19 S VET. The agreement between RBD-S SALUVET ELISA and INgezim^®^ COVID 19 S VET was almost perfect (*k* = 0.89), and ELISA COVID UNIZAR showed substantial agreement with LFIA COVID UNIZAR (*k* = 0.81) and RBD-S SALUVET ELISA (*k* = 0.74). The lowest kappa values were obtained between N SALUVET ELISA or WB and all other tests (*k* < 0.32).

**Table 3:** Test agreement (κ-values) between serological tests developed for cats (A) and dogs (B).

		**Tests based on S protein**						**Tests based on N protein**		
		**RC**	**RBD-S SALUVET ELISA**	**ELISA COVID UNIZAR**	**INgezim**^^®^^ **COVID 19 S VET**	**RBD-S SALUVET Western blot**	**LFIA** ^*^ **COVID UNIZAR**	**N-SALUVET ELISA**	**ID Screen**^^®^^ **SARS-CoV-2**	**N-SALUVET Western blot**
**(A)**										
Tests based on S protein	Reference criterium (RC)^*^	**1**	**0.85**	**0.89**	**0.88**	0.76	0.81	0.26	0.69	0.38
	RBD-S SALUVET ELISA	**0.85**	**1**	0.74	**0.89**	0.66	0.63	0.31	0.56	0.40
	ELISA COVID UNIZAR	**0.89**	0.74	**1**	0.82	0.76	0.81	0.26	0.69	0.38
	INgezim^®^ COVID 19 S VET	**0.88**	**0.89**	0.82	**1**	0.63	0.81	0.27	0.66	0.41
	RBD-S SALUVET Western blot	0.76	0.66	0.76	0.63	**1**	0.79	0.32	0.79	0.55
	LFIA^*^ COVID UNIZAR	0.81	0.63	0.81	0.81	0.79	**1**	0.28	0.71	0.41
Tests based on N protein	N-SALUVET-ELISA	0.26	0.31	0.26	0.27	0.32	0.28	1	0.30	0.63
	ID Screen^®^ SARS-CoV-2	0.69	0.56	0.69	0.66	0.79	0.71	0.30	**1**	0.39
	N-SALUVET Western blot	0.38	0.40	0.38	0.41	0.55	0.41	0.63	0.39	**1**
**(B)**										
Tests based on S protein	RC	**1**	0.55	0.21	**0.62**	0.50	0.10	0.15	0.17	0.32
	RBD-S SALUVET ELISA	0.55	**1**	0.25	**0.70**	0.58	0.17	0.32	0.24	0.31
	ELISA COVID UNIZAR	0.21	0.25	**1**	0.20	0.11	**0.79**	0.03	0.07	0.25
	INgezim^®^ COVID 19 S VET	**0.62**	**0.70**	0.20	**1**	0.61	0.20	0.42	0.39	0.19
	RBD-S SALUVET Western blot	0.50	0.58	0.11	0.61	**1**	0.04	0.20	0.14	0.58
	LFIA^*^ COVID UNIZAR	0.10	0.17	**0.79**	0.20	0.04	**1**	0.01	0.05	0.03
Tests based on N protein	N-SALUVET ELISA	0.15	0.32	0.03	0.42	0.20	0.01	**1**	0.07	0.21
	ID Screen^®^ SARS-CoV-2	0.17	0.24	0.07	0.29	0.14	0.05	0.07	**1**	0.29
	N-SALUVET Western blot	0.32	0.31	0.25	0.19	0.58	0.03	0.21	0.29	**1**

^*^Reference criterium: Virus seroneutralization assay and/or RT-qPCR.

LFIA, lateral flow immunoassay.

The highest *K*- values are marked in bold letters.

All assays, except for the N SALUVET ELISA, were strongly correlated (Table 4A) (Pearson *r* > 0.70 between ELISAs and Pearson *r* = 0.65 when ELISAs were compared with VNA).

**Table 4:** Correlation coefficients (Pearson *r*) between quantitative serological techniques for cats (A) and dogs (B).

		**Tests based on S protein**				**Tests based on protein N**	
		**VNA**	**RBD-S SALUVET ELISA**	**ELISA COVID UNIZAR**	**INgezim**^®^ **COVID 19 S VET**	**N-SALUVET ELISA**	**ID Screen**^®^ **SARS-CoV-2**
**(A)**							
Tests based on S protein	VNA	1	0.63^****^	0.62^****^	0.64^****^	−0.04	0.68^****^
	RBD-S SALUVET ELISA	0.63^****^	1	**0.85** ^****^	**0.89** ^****^	−0.08	0.75^****^
	ELISA COVID UNIZAR	0.62^****^	**0.85** ^****^	1	**0.94** ^****^	0.06	0.78^****^
	INgezim^®^ COVID 19 S VET	0.64^****^	**0.89** ^****^	**0.94** ^****^	1	0.36^**^	0.77^****^
Tests based on N protein	N-SALUVET ELISA	−0.04	−0.08	0.06	0.36^**^	1	0.06
	ID Screen^®^ SARS-CoV-2	0.68^****^	0.75^****^	0.78^****^	0.77^****^	0.06	1
Tests based on S protein	VNA	1	0.34^**^	0.23^*^	0.279^*^	−0.02	0.10
	RBD-S SALUVET ELISA	0.34^**^	1	0.60^****^	**0.88** ^****^	0.29^*^	0.04
	ELISA COVID UNIZAR	0.23^*^	0.60^****^	1	0.68^****^	0.12	0.11
	INgezim^®^ COVID 19 S VET	0.27^*^	**0.88** ^****^	0.68^****^	1	0.21^*^	0.19
Tests based on N protein	N-SALUVET ELISA	−0.02	0.29^*^	0.12	0.21	1	0.19
	ID Screen^®^ SARS-CoV-2	0.10	0.04	0.11	0.19	0.19	1

VNA, virus seroneutralisation assay. The highest correlations (Pearson *r* > 0.85) are marked in bold letters.

Significant statistical different: ^*^p < 0.05; ^**^p < 0.01; ^***^p < 0.001; ^****^p < 0.0001.

#### 3.1.3. Analytical specificity of RBD-S and N SALUVET ELISAs and INgezim^®^ COVID 19 S VET

**Figure 2:**
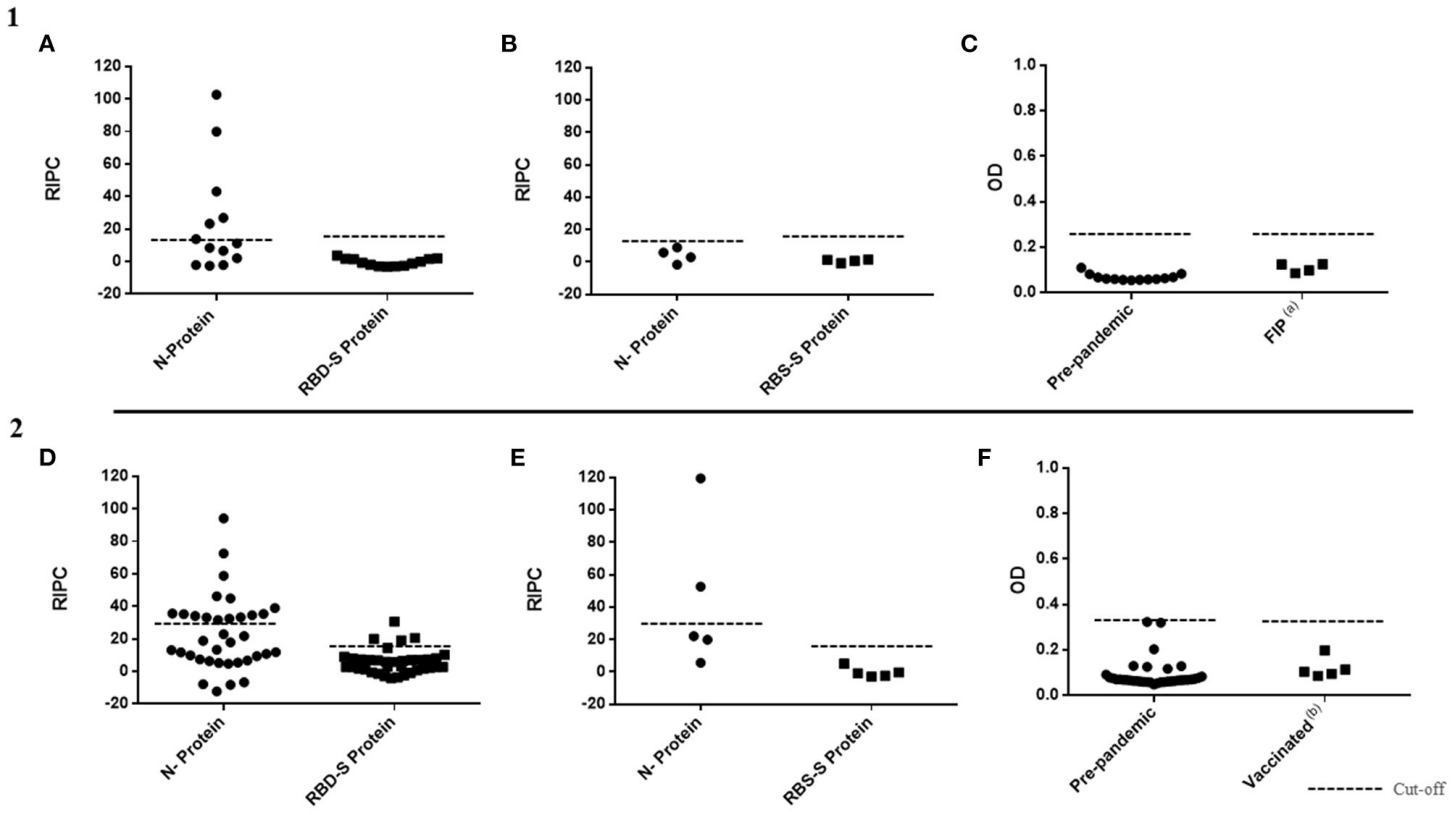
Analytical specificity of N SALUVET ELISA, RBD-S SALUVET ELISA and INgezim^®^ COVID 19 S VET developed for cats (1) and dogs (2). **(A)** Prepandemic cat sera analyzed by N and RBD-S SALUVET ELISAs; **(B)** sera from cats with feline infectious peritonitis (FIP) analyzed by N and RBD-S SALUVET ELISAs; **(C)** prepandemic and FIP sera analyzed by INgezim^®^ COVID 19 S VET; **(D)** prepandemic dog sera analyzed by N and RBD-S SALUVET ELISAs; **(E)** sera from dogs vaccinated with canine coronavirus analyzed by N and RBD-S SALUVET ELISAs; **(F)** prepandemic and sera from dogs vaccinated with canine coronavirus analyzed by INgezim^®^ COVID 19 S VET. RIPC, relative index percent; OD, optical density. (a) Cat sera from animals that died from feline infectious peritonitis (FIP). (b) Sera from dogs vaccinated with VANGUARD^®^ PLUS CPV/CV (Zoetis, New Jersey, USA).

The ASp of N and RBD-S SALUVET ELISAs and INgezim^®^ COVID 19 S VET was analyzed since these tests showed the best diagnostic performance for both N- and S- SARS-CoV-2 proteins and for both cats and dogs. When pre-pandemic cat sera were analyzed by RBD-S SALUVET ELISA and INgezim^®^ COVID 19 S VET all sera were considered negative (RBD-S SALUVET ELISA mean RIPC: 0.47, SD mean value: 0.12; INgezim^®^ COVID 19 S VET mean OD: 0.06, SD mean value: 0.01). No cross-reactions were noticeable when FIP-positive cat sera were tested by RBD-S SALUVET ELISA (mean RIPC: 0.67; SD mean value: 1.05) and INgezim^®^ COVID 19 S VET (mean OD: 0.10; SD mean value: 0.02) (Figures 2A, C). In contrast, six false-positive pre-pandemic cat sera were obtained with N SALUVET ELISA (RIPC mean value 23.80; SD mean value: 33.01) (Figure 2A). However, none of the sera from cats infected with FIP were positive when using the N SALUVET ELISA (RIPC mean 4.49; SD mean value: 5.78) (Figure 2B).

#### 3.1.4. Precision of RBD-S and N SALUVET ELISAs

All CV values of intra- and inter-plate repeatability were below 20% for RBD-S SALUVET ELISA and 97.7% for N SALUVET ELISA. The mean CV values for S-RBD-ELISA were 5.79 [standard deviation (SD) mean value: 4.14] for the intra-plate repeatability and 6.49 (SD mean value: 3.85) for the inter-plate repeatability. For N-ELISA, the intra-plate repeatability showed a mean CV value of 7.88 (SD mean value: 4.53), and the inter-plate repeatability showed a mean CV value of 10.43 (SD mean value: 4.04).

### 3.2. Serological tests developed for dogs

#### 3.2.1. Diagnostic performance of all serological test

The area under the curve (AUC) and DSe and DSp values are shown in Figure 1B and Table 2, respectively.

The S-RBD SALUVET ELISA showed the highest AUC (0.86; 95% confidence interval CI: 0.694–0.995). The cut-off was established for a RIPC > 15.85 with 81% DSe (95% CI: 0.482–0.977) and 87% DSp (95% CI: 0.752–0.953). INgezim^®^ COVID 19 S VET showed an AUC of 0.83 (95% CI: 0.628–1.027), followed by ELISA COVID UNIZAR (AUC: 0.81; 95% CI: 0.710–0.985).

The N SALUVET ELISA and ID Screen^®^ SARS-CoV-2 double antigen Multispecies ELISA showed AUCs of 0.55 (95% CI: 0.379–0.723 and 0.421–0.802, respectively). The N SALUVET ELISA cut-off was established for a RIPC > 26.34 with 60% DSe (95% CI: 0.234–0.832) and 65% DSp (95% CI: 0.503–0.783).

#### 3.2.2. Test agreement and correlation

The agreement between serological techniques in dogs is shown in Table 3B and is expressed as *Kappa (k)* values. INgezim^®^ COVID 19 S VET and RBD-S SALUVET ELISA showed the highest agreement between them (*k* = 0.73*)* and with reference criterium (*k* = 0.62 and 0.55, respectively). Substantial agreement was observed between ELISA COVID UNIZAR and LFIA COVID UNIZAR *(k* = 0.79), and the agreement was lower when these tests were compared with other techniques. Tests based on N-protein showed little or no agreement (*k* < 0.42), except for RBD-S and N SALUVET WBs, which showed substantial agreement (*k* = 0.58).

As shown in Table 4B, the highest correlations were accomplished between tests based on S-protein and low or no correlation between N protein-based tests. The highest correlation was observed between INgezim^®^ COVID 19 S VET vs. RBD-S SALUVET ELISA (Pearson *r* = 0.88) and ELISA COVID UNIZAR (Pearson *r* = 0.68). There was a remarkably weak correlation between VNA and the other tests.

#### 3.2.3. Analytical specificity of RBD-S and N SALUVET ELISAs and INgezim^®^ COVID 19 S VET

Thirty-six prepandemic dog sera and five sera from vaccinated dogs were analyzed by RBD-S SALUVET ELISA, N SALUVET ELISA and INgezim^®^ COVID 19 S VET (Figures 2D–F). Thirty-two of 36 prepandemic sera were negative by RBD-S SALUVET ELISA (mean RIPC: 5.75, SD mean value: 7.46). In turn, 15 out of 36 prepandemic sera were positive when analyzed by N SALUVET ELISA (Figure 2D). All prepandemic sera were negative by INgezim^®^ COVID-19 S VET (mean OD: 0.09, SD mean value: 0.06) (Figure 2F).

Likewise, when sera from vaccinated dogs were analyzed, cross reactions were observed in two out of five sera with N SALUVET (RIPC mean 31.09, SD mean 38.84) (Figure 2E). RBD-S SALUVET ELISA (RIPC mean 1.24, SD mean 4.67) and INgezim^®^ COVID 19 S VET (OD mean 0.11, SD mean 0.04) (Figures 2E, F) did not show cross-reactivity.

#### 3.2.4. Precision of RBD-S and N SALUVET ELISAs

When intra- and inter-plate repeatability of RBD-S and N SALUVET ELISAs were tested, 96 and 88% CV values were below 20%, respectively. The mean CV values for RBD-S SALUVET ELISA were 9.49 (SD mean value: 5.52) for the intra-plate repeatability and 4.37 (SD mean value: 4.57) for the inter-plate repeatability. For the N SALUVET ELISA, the intra-plate repeatability showed a mean CV value of 7.17 (SD mean value: 6.52), and the inter-plate repeatability showed a mean CV value of 10.84 (SD mean value: 9.58).

## 4. Discussion

A low number of serological tests have been developed for the detection of specific IgG antibodies against SARS-CoV-2 in pets (7, 14) compared with the wide battery of assays available for humans (20). Moreover, these tests have been employed in epidemiological studies, but an exhaustive validation procedure is lacking, likely due to the absence of a panel of well-coded reference sera (12, 21). Herein, six serological tests were developed (three different ELISAs, two Western blots and one LFIA test). Next, a comparative study including all in-house assays and two commercial ELISAs was performed for the first time in pets. The results obtained allowed us to select the most accurate serological tests for cats and dogs.

The main pillars of the validation procedure relied on the restrictive criteria employed to classify sera as positive or negative (VNA and/or RT-qPCR results) and on the cat and dog reference sera panels employed to analyse ASp and diagnostic performance. The validation process followed the recommendations for the standardization of diagnostic techniques from the World Organization for Animal Health (OIE) (19).

Our restrictive reference criteria based on VNA and RT-qPCR results prioritized the DSp of the tests evaluated. The VNA was considered the reference test in previous studies that developed serological tests to detect anti-SARS-CoV-2 specific antibodies in pets (13, 22). The DSp of this assay relies on the detection of specific antibodies that neutralize the virus, but DSe might be dependent on the circulating virus variant. In humans, it has been reported that the delta variant (B.1.617.2) can escape from specific antibodies directed against the alpha variant (B.1.1.7) (23). Moreover, it is also unknown how long viral particles can be detected in infected animals by means of RT-qPCR, so that the time elapsed between a positive RT-qPCR result and seroconversion should also limit DSe. These arguments could explain the higher Se detected with conventional tests based on S protein compared with VNA (24). To circumvent these limitations and in the absence of a more appropriate reference test, first, we tried to detect the virus by RT-qPCR. Next, we searched for specific anti-SARS-CoV2 antibodies in sera collected 21 days later by VNA. In a previous work, the kinetics of virus detection was investigated in one cat and three dogs that showed a RT-qPCR positive result and three animals revealed specific IgGs shortly after or simultaneously to virus shedding, whereas the remaining animal showed a low viral load and seroconverted 21 days after SARS-CoV-2 detection (8). Accordingly, a serum was considered as a reference positive serum when the animal of origin showed either a RT-qPCR positive result, a VNA positive result or both RT-qPCR and VNA positive results, considering 1/20 VNA titer as the cut-off. This conservative criterion could be modified in the future once validated tests are available and can be employed as reference tests.

The panels of sera employed were composed of a proper number of cat and dog sera to determine ASp as well as diagnostic performance. Regarding ASp, prepandemic sera as well as sera from animals either infected with or vaccinated against other coronaviruses were analyzed. Both panels of sera are essential for accurate assay validation, as demonstrated by a readjustment of RBD-S SALUVET ELISA needed for dog sera and discussed below. These serum panels were not employed systematically in all previous studies, and the number of positive sera from pets was usually very low. Wernike et al. (14) validated a multispecies ELISA based on RBD-S fragments, but only three positive animals were analyzed, and sera from animals infected with other coronaviruses were not used to confirm the ASp. Similarly, Zhao et al. (13) analyzed only 12 positive cat sera and two positive dog sera out of a total of 500 cat or dog sera by ELISA that used SARS-CoV-2 S1 and RBD-S proteins as antigens.

Tests based on the RBD-S fragment or S protein showed the best diagnostic performance when cat and dog sera were analyzed. Moreover, tests developed for cats seem to be more accurate than those developed for dogs. RBD-S SALUVET ELISA together with ELISA COVID UNIZAR and INgezim^®^ COVID 19 S VET developed for cats showed the best results in terms of DSe and DSp. Thus, these tests could be equally employed in SARS-CoV-2 surveillance in cats with comparable results. On the other hand, RBD-S SALUVET ELISA and INgezim^®^ COVID 19 S VET showed the highest DSe and DSp values with dog sera. However, diagnostic performance values higher than 90% were not obtained with any of the tests evaluated, and TG-ROC analysis results indicated that readjustment of these tests cannot improve their diagnostic performance in dogs. Our results agree with previous studies that developed ELISAs based on the S-protein for pets and showed that the S-protein had the best diagnostic performance when different methods were compared with VNA (13, 14). Wernike et al. (14) developed a multispecies ELISA based on the RBD-S domain for cats with 100% DSp and 98.31% DSe with a cut-off of OD > 0.3. The ELISAs using SARS-CoV-2 S1 and RBD proteins, which were applied by Zhao et al. (13), showed a strong correlation with each other (Pearson *r* = 0.95), and both correlated well with VNA (Pearson *r* = 0.87). These authors also discarded the use of N-protein in the serodiagnosis of SARS-CoV-2 in pets. Similarly, Barua et al. (11) reported low agreement between ID Screen^®^ SARS-CoV-2 ELISA and seroneutralization when dog sera were tested. These results agree with the data obtained herein since N-based assays did not show acceptable DSe and DSp values supported by the ASp results discussed below. In both pet species, N-protein ELISAs showed a poor correlation with reference tests and RBD-S ELISAs (RBD-S SALUVET ELISA, ELISA COVID UNIZAR) and whole S-protein (INgezim^®^ COVID 19 S VET). Our results are supported by Zhao et al. (13), who reported the highest correlation between S-based ELISA and seroneutralization and disease severity.

The serological tests compared in the present work were based on spike (S) and nucleocapsid (N) viral proteins. To date, all the immunoassays developed for animals have employed RBD-S protein, whole S-protein (13, 14) or N-protein (7, 13). The spike protein is trimeric and can be cleaved by host proteases into the S1 and S2 subunits. The S1 subunit has a unique region called the receptor-binding domain (RBD), which is used by the virus to enter the host cell through recognition of the angiotensin-converting enzyme 2 receptor. Thus, the S protein plays a crucial role in the entry of the virus into the host cell, and variability of the RBD domain among different coronaviruses has been reported. However, the N-terminal domain (NTD)-N protein does not bind to the receptor, and it is known that nucleocapsid proteins of coronaviruses are relatively conserved and that the presence of cross-reactive epitopes in the N-proteins within a particular subgroup and among different subgroups raises potential concerns in serological assays based on N proteins, with significant antigenic cross-reactivity (25, 26). Cross-reactivity has been suggested between SARS-CoV-2 and feline coronavirus type I N proteins (13), canine enteric coronavirus and canine respiratory coronavirus (27–29). In contrast, Zhao et al. (13) did not detect cross-reactions with antibodies against feline coronavirus in cats. All this evidence could explain the detection of cross reactors when the N protein is employed in serological tests and the absence of cross reactors when the S protein is used (30). These previous results are also supported by our findings. We observed cross-reactions when cat and dog sera were obtained prior to the COVID-19 pandemic, and dog sera from vaccinated dogs against other coronaviruses were analyzed with N SALUVET ELISA, which contrasts with better results obtained with tests based on the S protein. Similar observations have been reported for serological tests employed in humans since S-based assays showed high specificity, contrary to N-based tests, regardless of the IgG isotype targeted. Although the S protein seems to be a specific marker of SARS-CoV-2 in humans (31), this antigen showed no cross reactions with prepandemic sera and sera from cats infected with other coronaviruses, and the detection of false reactors cannot be ruled out. In fact, a few prepandemic dog sera showed a positive result when tested with RBD-S SALUVET ELISA. Thus, the existence of a few cross reactors suggests that a readjustment of the cut-off of this assay should be considered to the detriment of DSe (RIPC cut-off = 31; DSe = 60%; DSp = 95%). Similarly, previous authors found a high rate of SARS-CoV-2 S protein seropositivity with prepandemic sera from pets (32) and highlighted the need for a better understanding of the prevalence and crossover potential of wild coronaviruses. The existence of false-positive reactors could be explained by the presence of other infectious agents, multiple coronavirus infections and the existence and circulation of a SARS-related virus containing the S or RBD-S sequence (32).

Our study supports the employment of ELISAs based on the S protein for serosurveillance purposes in pets since they can help to elucidate virus transmission and other epidemiological gaps (8). However, the use of tests that employ cut-offs based on OD values should be supported by repeatability data, as done for RBD-S and N SALUVET ELISAs and these preliminary cut-off estimations should be re-validated during the last stage of the validation process of serological tests [stage of monitoring and maintenance of validation criteria according to OIE guidelines, (19)]. The virus seroneutralisation assay would be adequate as a confirmatory technique to be employed in a limited number of samples due to the need for specialized biocontainment facilities (Biosafety Level 3) and considering that seroneutralization is not an automated technique for large screening. Regarding the WBs, further refinement of these techniques seems to be required to increase their DSe and the agreement between these assays and their corresponding ELISAs. Finally, the lateral flow immunoassay test (LFIA) is a simple, quick, reliable, and easy-to-use on-site tool that could be used for SARS-CoV-2 diagnosis in cats since it is very specific, but its DSe is limited. The use of a calibrated electronic strip reader would allow obtaining an objective determination of the LFIA results, avoiding misinterpretation of the results.

It should be noted that relevant gaps in knowledge limit the interpretation of serological results. First, it remains unknown how long specific antibodies elicited against SARS-CoV-2 persist in pets. It is well-known that serological tests can detect past infections after the immune system has successfully cleared the infection or after the onset of illness [(20); https://www.cdc.gov/coronavirus/2019-ncov/lab/resources/antibody-tests-guidelines.html]. However, longitudinal studies are needed to understand when pets seroconvert, and the kinetics of antibody levels. Experimental studies reported seroconversion in cats at 7–12 days postinfection (dpi) and 14 dpi in dogs by means of VNA (33), but the agreement between VNA and ELISA was not investigated. Other issues to be considered are the individual variability detected in a preliminary screening performed in 15 animals that analyzed sequential serum samples that might be influenced by the circulating virus variant (34).

In conclusion, the present comparative study demonstrated that ELISA tests based on the S-protein appear to be the most accurate tests in cats and dogs. These tests showed better diagnostic performance in cats, and tests should be improved for dogs. Moreover, there is a need to check the analytical Sp of any serological test employed to avoid false-positive reactors and for a more accurate assay validation. Accordingly, these results set the basis for the selection of the best diagnostic approach for each species for surveillance purposes. The employment of the most accurate test will help to better elucidate the possible contribution of companion animals to SARS-CoV-2 transmission, the risk they might pose to humans, in addition to helping monitor future changes in disease pattern. Further strategies should attempt to improve their diagnostic performance since there is room for further improvement with particular interest in tests developed for dogs.

## Data availability statement

The raw data supporting the conclusions of this article will be made available by the authors, without undue reservation.

## Ethics statement

The animal study was reviewed and approved by Animal Experimentation Committee-Complutense University of Madrid. Written informed consent was obtained from the owners for the participation of their animals in this study.

## Author contributions

GÁ-G, LO-M, and JR-C proposed and designed the study. CD-D, SV-S, MD, MV, PG-M, AB, and SM performed the serological analyses. GM, RC, and AM collected the serum samples. WV provided the proteins. CD-D, GÁ-G, JR-C, and LO-M analyzed the results and drafted the manuscript, interpreted the results, and were assisted by GM, SV-S, MD, MV, PG-M, AB, SM, RC, AM, and WV. All authors read and approved the final manuscript.

## Supplementary material

Supplementary Figure Cat and dog sera analyzed by RBD-S and N SALUVET Western blots.
