# Supplementary figures and images for: A comparative study of eight serological methods shows that spike protein-based ELISAs are the most accurate tests for serodiagnosing SARS-CoV-2 infections in cats and dogs
Source: Front Vet Sci. 2023 Jan 26;10:1121935. doi: 10.3389/fvets.2023.1121935 (PMC9909348; doi:10.3389/fvets.2023.1121935)

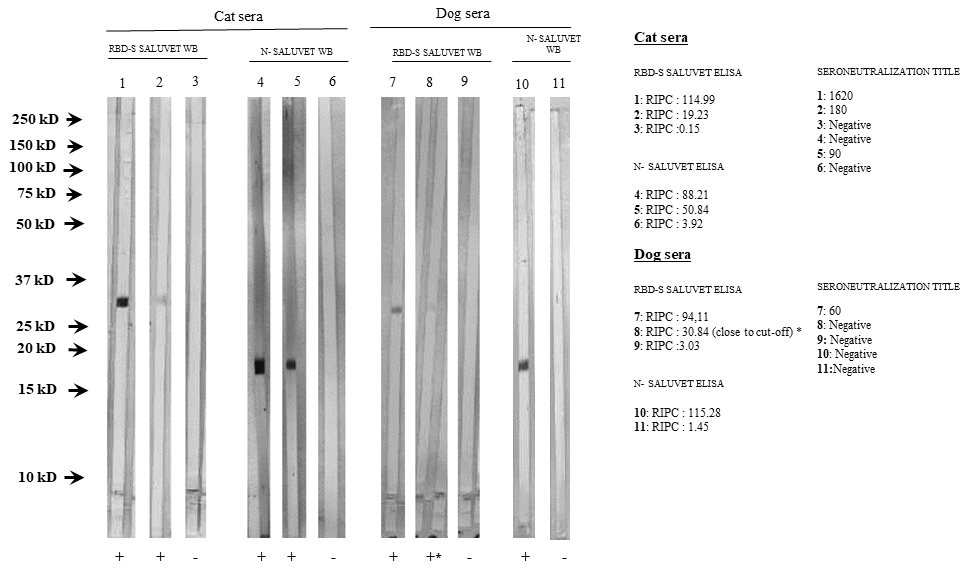

Supplement: Supplementary Figure — Cat and dog sera analyzed by RBD-S and N SALUVET Western blots. [file Image_1.TIF]
